# Impact of temperature on the virulence of *Streptococcus agalactiae* in Indonesian aquaculture: A better vaccine design is required

**DOI:** 10.14202/vetworld.2024.682-689

**Published:** 2024-03-22

**Authors:** Angela Mariana Lusiastuti, Achmad Suhermanto, Bernadetta Rina Hastilestari, Suryanto Suryanto, Mira Mawardi, Desy Sugiani, Dewi Syahidah, Putu Eka Sudaryatma, Domenico Caruso

**Affiliations:** 1 Research Center for Veterinary Sciences, National Research and Innovation Agency, KST BRIN Soekarno Cibinong Bogor, 16911, Jawa Barat, Indonesia; 2 The Marine and Fisheries Polytechnic Karawang, The Ministry of Marine Affairs and Fisheries Indonesia; 3 Research Center for Genetic Engineering, National Research and Innovation Agency, Indonesia; 4 Research Center for Fisheries, National Research and Innovation Agency, Indonesia; 5 Main Center for Freshwater Aquaculture – The Ministry of Marine Affairs and Fisheries, Jl. Selabintana No. 37, Selabatu, Kec. Cikole, Kota Sukabumi, Jawa Barat 43114, Indonesia; 6 Fish Quarantine, The Ministry of Marine Affairs and Fisheries; 7 ISEM, Univ. Montpellier, CNRS, EPHE, IRD, Montpellier, France

**Keywords:** adaptation, microbes, pathogen, temperature, virulence

## Abstract

Due to their poikilothermic nature, fish are very sensitive to changes in temperature. Due to climate change, the average global temperature has increased by 1.5°C in the last century, which may have caused an increase in farmed fish mortality recently. Predictions using the model estimate that a 1°C increase in temperature could cause 3%–4% and 4%–6% mortality due to infectious diseases in organisms living in warm and temperate waters, respectively. There is a need to determine whether there is a relationship between increasing environmental temperature and disease virulence. This review examines the influence and impact of increasing temperatures due to climate change on the physiology and pathogenicity of *Streptococcus agalactiae*, which causes streptococcosis in tilapia and causes significant economic losses. Changes in the pathogenicity of *S. agalactiae*, especially its virulence properties due to increasing temperature, require changes in the composition design of the fish vaccine formula to provide better protection through the production of protective antibodies.

## Introduction

According to the report of Fish Site [1], Streptococcosis caused by *Streptococcus agalactiae* has attacked 43 farms in four regions of South America and is classified as a national emergency. This is a serious threat because it has the potential to be economically detrimental, leading to the loss of livelihoods of farmers due to fish mortality exceeding 50% [2].

Aquaculture is Indonesia’s mainstay for overcoming poverty because it supports food security [3]. Indonesia is a middle-income country with a variety of aquaculture production methods, including small-scale natural production such as rice fish cultivation, semi-extensive and extensive pond cultivation, both fertilized and unfertilized, intensive cultivation in floating net cages in lakes or offshore, or super-intensive shrimp cultivation [4]. Developing countries, such as Indonesia, support 90% of global aquaculture production and play an important role in global food security and poverty alleviation under the 2030 Sustainable Development Goals. However, warmer global temperatures appear to threaten aquaculture production due to the increasing incidence of new infectious diseases (emerging infectious diseases) such as, cyprinid herpesvirus 3 (CyHV-3) which is responsible for koi herpesvirus disease (KHVD) is associated with increasing water temperatures. Betanodavirus, known as nerve necrosis virus (NNV), causes encephalopathy and retinopathy in marine showing increased virulence when exposed to higher water temperatures [5]. Some very concerning diseases, such as Edwardsiellosis disease in catfish, streptococcosis in tilapia, and acute hepatopancreatic necrosis disease in shrimp, become epizootic when the temperature increases [6–9].

Reverter *et al*. [10] examined 272 studies in the literature and found a link between temperature and mortality in aquatic animals due to bacterial infection. According to Leung and Bates [11], an increase in disease outbreaks in aquatic animals in subtropical countries is associated with an increase in temperature and higher nutrient content in the waters. Furthermore, linear mixed models showed that pathogens such as Aeromonas spp., Edwardsiella spp., *Flavobacterium columnare*, Lactococcus spp., Streptococcus spp., and Vibrio spp. cause increased mortality of aquatic animals by 2.82%–4.12% as the temperature increases by 1°C in tropical countries.

Stress due to rising temperature extremes in the environment can damage the immune system in aquatic animals and make them more susceptible to infectious diseases [12]. However, whether higher temperatures can increase that the virulence of pathogens remains controversial [13].

Rising temperature tends to accelerate the rate of replication, virulence, life cycle longevity, and pathogen transmission among several species of finfish and shellfish [14]. According to Sae-Lim *et al*. [15], new disease outbreaks due to warmer water temperatures would appear more frequently, and the increased temperature may increase the impact of the emergence of epizootic diseases in aquaculture and cause serious economic losses. In addition, water temperature is an important factor that affects fish. Fluctuations in water temperature can affect dissolved oxygen levels, salinity, and pH, both individually and synergistically [16]. Neuman *et al*. [17] reported that the pathogen *Vibrio* spp. grows well and thrives in warm temperatures.

This review examines the influence and impact of increasing temperatures due to climate change on the physiology and virulence properties of *S. agalactiae* as well as the possible need to change the composition of existing vaccines to provide more effective protection.

## Rising Temperature

In the past 30 years, the temperature has gradually increased, and the 2000s have been the warmest decade. The increase in temperature is caused by greenhouse gases that trap heat and prevent it from entering the atmosphere of the Earth. Despite efforts to reduce greenhouse gas emissions, temperatures continue to increase. The climate is becoming warmer, leading to more intense rainstorms and blizzards, higher rainfall than usual, and storms around the world can occur more often and become stronger. Hurricanes can cause flooding, damage buildings, roads, and other structures, damage crops, and endanger human life [18]. The increase in global temperature of 2°C poses a high risk to the world, leading to the melting of polar ice, the bleaching of coral acidification of the sea, and the migration of fish species, so that more than 50% of catches in the tropics and Antarctica will decrease.

Sea levels in Indonesia’s coastal areas are rising by 3–5 mm per annum, creating conditions for flooding and seawater intrusion. Fifty percentages of the total biodiversity are threatened and 80% of coral reefs are damaged due to rising sea surface temperature. Climate change leads to a decline in the abundance of fish larvae due to changes in fish habitats, especially fish that Indonesians rely on for food and livelihood. Fish mortality, especially in tilapia, has also been widely reported when environmental temperature increases above normal. In agriculture, crop yields are expected to decrease by 25% by 2050. Rainfall patterns have changed as surface temperatures in Indonesia have increased by 0.2°C–0.3°C per decade.

Reduced rainfall during critical periods increases the risk of drought in Java, Bali, and Nusa Tenggara, whereas increased rainfall during wet periods increases the risk of flooding in Sumatra and Kalimantan. El Nino/La Nia events will become stronger and more frequent, leading to an increase in drought and/or flooding and reducing food production. It is also inevitable that human health impacts. More frequent and severe heat waves, floods, extreme weather events, and long-lasting droughts lead to increased injuries and diseases such as malaria, dengue fever, diarrhea, respiratory distress, and death.

## Effect of Climate Change on Micro-organisms

Biodiversity loss, that is, climate-induced extinctions of animals and plants, has often been studied, discussed, and published [19]. On the other hand, the existence of micro-organisms due to climate change has been less discussed and published, possibly because they are micro-organisms that can only be seen through a microscope; therefore, they are not easy to study [20]. Microbial abundance and diversity play an important role in ecosystems that support life in the biosphere, thus ensuring a healthy and balanced ecosystem [21]. They significantly contribute to carbon sequestration and, therefore, play an important role in the global carbon cycle. However, micro-organisms also contribute to greenhouse gas emissions through heterotrophic respiration (CO_2_), methanogenesis (CH_4_), and denitrification (N_2_O) processes [22].

The global average temperature has increased from 0.5°C to 0.7°C and is expected to continue increasing by 0.3°C per decade [23, 24]. According to Qin *et al*. [25], by the end of the 21^st^ century, global temperatures will increase by 1.1°C–6.4°C. This is supported by the opinion of Sun *et al*. [26] that the wet bulb globe temperature plays a role in the creation of heat stress due to dry air temperature and humidity. Therefore, climate change and global warming affect the microbial community sooner or later because they can alter the interactions between microbes and their hosts [27].

Temperature, oxygen content, pH, and CO_2_ can affect the local microbial community, biomass, abundance, and diversity [28]. Deeper changes occur in microbial metabolism and their respiratory system, which indirectly affect microbial ecosystem chains in the environment, such as nitrification, nitrogen fixation, denitrification, and carbon cycle. The physiology of the host changes due to high temperatures, the speed of chemical and enzymatic reactions, diffusion rate, membrane fluidity, and protein structure in the body’s metabolic processes [29].

For example, Antarctica and the Arctic have been exposed to increasing temperatures in the past 6 months, which has led to an increase in cyanobacteria and blooms in different lakes and reservoirs. Blooming cyanobacteria produce various types of neurotoxins, hepatotoxins, and dermatoxins that are lethal to birds and mammals [30]. They can hurt people if they migrate and carry these poisons.

## Adaptation Mechanism

In complex heterogeneous environments or despite ecological disturbances, microbes can adapt to fluctuating environmental conditions through cascades of cellular and molecular systems. Microbes are able to develop mechanisms to cope with environmental changes and maintain their role in the ecosystem.

Micro-organisms always adapt dynamically to respond to a fluctuating environment; therefore, this process often takes a long time. A changing environment can trigger a microbial stress response in which microbes try to stay alive rather than focus on growth [31]. According to Tan *et al*. [32], microbes adapt through natural selection, genetic recombination, horizontal gene transfer, and DNA damage repair. This finding is similar to that reported by Brooks *et al*. [33], who found that microbial populations try to survive pressure due to environmental changes by adapting within a certain period of time by inducing cellular, genetic, and morphological modulations.

Microbial adaptation involves the expression or repression of many genes. This mechanism alters the properties of intracellular, extracellular, and surface micro-organisms [34]. High environmental temperature is an environmental change that triggers microbe stress. At high temperatures, microbes, such as *Escherichia coli* and *Zymomonas mobilis*, have genes responsible for their growth through membrane transport mechanisms, energy metabolism, DNA repair, tRNA modification, membrane stabilization, and cell division [35]. Microbes adapt to rising temperatures using sensory biomolecules called thermosensors, which can activate a heat shock response and are expressed by two types of proteins, namely chaperons, and proteases [36]. In addition to temperature, other extreme environmental conditions such as ultraviolet (UV) irradiation, high pH, and salinity cause microbes such as *Vibrio parahaemolyticus* to form biofilms [37, 38]. According to Wolska *et al*. [39], this biofilm is one of the efforts of microbes to adapt to exogenous stress. Several types of bacteria, such as pathogenic Salmonella, use biofilm formation to survive in extreme environments and can increase their virulence [40]. In addition to temperature, biofilm formation is influenced by other environmental conditions such as pH, glucose, and water activity [41].

Adaptation can rapidly increase due to stress tolerance [42]. Adaptation refers to the ability of microbes to cope with stress in a short time. Micro-organisms undergo long-term and short-term adaptation through different mechanisms. Genome mutations occur as a reaction to stressful environments by increasing product yields or strain growth rates [43].

Many microbial adaptations are required to survive under different environmental conditions, as described in Table-1 [44–50]. High temperatures accompanied by various toxic substances can affect micro-organism survival. Microbes can adapt quickly to substrates or modify existing substrates around them, which can be passed on to their offspring. Microbial tolerance is a complex trait that is not well controlled by one gene or several genes [51–53]. Thus, it is common for microbes to cope with environmental stress by adapting to low to high levels of stress. Antimicrobial drug resistance is a form of adaptation of microbes to their environment [54, 55]. Cell membranes and genetic products produced by bacteria can change with temperature, pH, pressure, and salinity [56]. These changes are manageable and can be passed on to the next generation. *V. parahaemolyticus* adapts to changing environmental conditions by changing into different cell types (swarm cells) to maintain optimal growth and motility [57, 58]. This adaptation involves a number of genes involved in their expression and repression.

**Table-1:** Adaptation strategies of microorganisms in fluctuating environments [44].

Microorganisms	Microorganism in extreme environments	Mechanism of adaptation	References
*Acidithiobacillus ferrooxidans, Halarchaeum acidiphilum, Metallosphaera sedula, Thiobacillus prosperous, Acetobacter aceti*	Acidophile (pH 0.5 to 5)	Efficient efflux system, acid tolerant membrane, proton exclusion and secondary transporters driven by protons	[45, 46]
*Halorhodospira halochloris, Thiohalospira alkaliphila, Bacillus firmus, Natronomonas pharaonis*	Alcaliphile (pH 8.5 to 11)	Efficient proton uptake system mediated by antiporters of the membrane, OH- ion	[45]
*Psychrobacter adeliensis, P. aestuarii, P. alimentarius, P aquimaris, P. luti, P glacincola*	Psychrophile (–10°C–40°C)	Synthesis of unsaturated fatty acids to prevent the decrease of membrane fluidity reduces the size of the cell and elevates cellular water in an ordered fashion	[47-49]
*Serratia ureilytica*	Thermophile (20°C–54°C)	Modified vegetative cells into resistant structures	[50]

## *S. agalactiae* Case

In 2015, 146 people in Singapore fell ill after eating a local dish with a traditional recipe of raw freshwater fish [59]. The patient was diagnosed with group B streptococcus (*S. agalactiae*) (GBS) *S. agalactiae* ST 283 poisoning. Barkham, a human doctor in Singapore, discussed GBS *S. agalactiae* ST 283, which is an invasive GBS in humans that was previously unknown to be transmitted through food and very rarely occurs in healthy adults [59]. Why does this happen? What is wrong with *S. agalactiae*? Did the source of *S. agalactiae* come from fish? In contrast, according to Barsøe *et al*. [60], fish vaccination is very important for the prevention of diseases. Therefore, *S. agalactiae* vaccine is required to prevent streptococcosis in tilapia. Can zoonotic potential bacteria be used as a preventive strategy against related pathogens?

Streptococcosis in aquaculture causes annual losses worldwide, for example, in 2000 and 2008, the losses were USD 150 million and USD 250 million, respectively [61]. Streptococcosis has been reported in many marine and freshwater fish species, including rainbow trout (*Onchorrynchus mykiss*), yellow tail (*Seriola quinqueradiata*), Asian seabass (*Lates calcarifer*), and Nile tilapia (*Oreochromis niloticus*). The annual loss due to streptococcosis in Iran appears to be even more severe at 30% due to predisposing factors such as poor health management and high ambient temperature [62]. Therefore, *S. agalactiae* is indeed present in fish.

*S. agalactiae* is the main cause of death in tilapia culture worldwide, particularly in Indonesia [63]. Tilapia streptococcal disease was originally caused by GBS, a virulent disease that also affects cattle and humans. In Indonesia, *S. agalactiae*-induced tilapia disease has several serotypes that affect juvenile and rearing tilapia [64]. Indonesian *S. agalactiae* possesses two types of β-hemolytic and non-hemolytic bacteria and can also be divided into capsulated and non-capsulated. *S. agalactiae* β-hemolytic has the capability to hydrolyze more sugars. β-hemolytic type bacteria have different characteristics when cultured on brain heart infusion agar media, namely, thick colonies, rather light-colored, slimy, and easy to harvest, whereas non-hemolytic type bacteria tend to be rather thin, transparent in color, sticky, and difficult to harvest. Studies on streptococcal on tilapia culture at the Cirata Reservoir in West Java, Indonesia, which were characterized using multilocus sequence typing (MLST), identified non-hemolytic *S. agalactia*e as the causative agent [63]. The results of this research prove that *S. agalactiae* in fish belongs to GBS, which is categorized as dangerous to humans. The non-hemolytic group possessed the following six virulence genes (*hylB, bibA, fbsA, fbsB, gap*, and *cfb*), while the hemolytic group possessed seven virulence genes (*cylE, hylB, bibA, PI-2b, fbsA, fbsB*, and *gap*) [65]. The number of *S. agalactiae* virulent genes is positively correlated with streptococcosis pathogenicity in tilapia. Syuhada *et al*. [66] reported that *S. agalactiae* serotypes Ia ST7 and III ST283 currently exist in cultured fish in Malaysia. Serotype III ST283 has the *lmb*, *scpB*, *pavA*, *fbsB*, *cyl*, *bca*, *cspA*, and *bac* genes and tends to cause acute infections in red hybrid tilapia.

MLST research revealed that *S. agalactiae* had cladogenesis. Separation of *S. agalactiae* from the phylogenetic tree to form sequence type (ST) 261, separate from the bovine clad and the human clad [67]. The separation of these species was due to the occurrence of polymorphism (single-nucleotide polymorphism [SNP]) in the *S. agalactiae* genome, namely, the presence of two bases that changed base 24 and base 167. A new base substitution occurred at base 24, whereas a base difference occurred at base 167. SNP mutations are probably caused by an increase in temperature due to global warming. In tilapia (*O. niloticus*), high water temperature increases the severity of streptococcosis. The high mortality rate in tilapia cultivated in cages in Lake Sentani, Papua [68], may also be due to the influence of temperature. Due to global warming, pathogenic bacteria cause increased exposure to humans. The number of SNPs may be increasing with increasing global temperature. SNPs are mutations that can cause increased pathogenicity due to changes in protein–protein interactions. Global warming, as an environmental trigger, increases replication errors or DNA damage, resulting in point mutations that can increase the rate of mutation. *V. parahaemolyticus* experienced mutations in the thermolabile hemolysin gene (*tlh*) due to increased temperature at seven high-frequency mutation points (A180G, T552G, G657T, T858C, C1062T, A1137G, and T1179C) [69]. *V. parahaemolyticus* adaptation due to climate change increased its virulence over time. As global temperatures increase, mutations in the thermolabile hemolysin (*tlh*) gene marker also increase. These results suggest that environmental isolates are adapting to a warming environment and becoming more pathogenic.

Phuoc *et al*. [70] conducted a challenge test on tilapia by comparing the virulence of several isolates of GBS ST 283 from GBS CC552, which had adapted to fish, and ST651 from CC103, which came from humans. This study shows that the severity and geographic extent of streptococcal outbreaks in tilapia are increasing due to changes in water conditions due to global warming, environmental pollution, and increasing acidity of water. The survival of tilapia was highest at a water pH of 7.5 and a water temperature range of 18°C to 20°C, indicating that tilapia can survive at a pH of 7.5. Microbes are able to adapt to different environments due to their genetic composition and arrangement. That’s what happened to *S. agalactiae*, whose adaptive behavior makes *S. agalactiae* virulent and deadly. Zhao *et al*. [71] demonstrated that high temperatures can change the gene expression of *S. agalactiae* and that tilapia become more susceptible to *S. agalactiae* infection in terms of the host.

**Figure-1:**
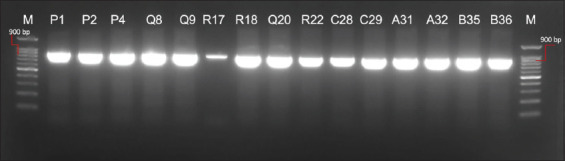
Virulence gene *cfb* of *Streptococcus agalactiae* type 1a and 1b. P1, P2, P4 = Papua isolate at 30°C; Q8, Q9, Q20 = Papua isolate Papua at 32°C; R17, R18, R22 = Papua isolate at 34°C; C28, C29 = Java isolate at 34°C; A31, A32 = Java isolate at 30°C; B35, B36 = Java isolate at 32°C.

Changes in environmental temperature from low to high temperature (35°C) modulate the rate of enzymatic activity and affect the transcription of virulent genes. The hemolytic activity of GBS increased fivefold, followed by an inflammatory response with upregulation of cyclooxygenase-2, *IL-1β*, and *TNF-α* genes between 6 and 96 h post-infection. This research is supported by Liao *et al*. [72], who stated that temperature, UV index, and rainfall were positively correlated with streptococcosis infection and caused an increase in tilapia by more than 50%, especially at temperatures above 27°C, atmospheric pressure lower than 1005.1 hPa, and UV index above 7.2. This finding that *S. agalactiae* becomes more virulent and deadly is also supported by Lam *et al*. [73], who reported that increasing temperature also causes changes to DNA topology, arrangement, RNA metabolism, activity, and protein processing. An increase in temperature affects DNA supercoiling, which is involved in the expression of virulence coding gene. Moreover, Lusiastuti *et al*. [74] explained that the characteristics of the Papua isolate of *S. agalactiae* in 2013 showed a negative CAMP (Christie, Atkinson, Munch, Peterson) test or *cfb* gene, but in 2023, the same *S. agalactiae* isolate showed a positive CAMP test. Meanwhile, the Java *S. agalactiae* isolate remained positive (Figure-1). It appears that increasing the temperature can change the virulence properties in anticipation of defending itself to survive in its environment.

Changes in the virulence of *S. agalactiae* with increasing global temperature raise the question as to whether it is part of the adaptation process. According to Habig *et al*. [75], genes that encode virulence factors can adapt to changes in the host environment. Repetition-induced point mutations or DNA methylation can increase the mutation rate in some species. Virulence factors in genome compartments susceptible to mutation may confer a higher rate of evolution for these genes. Consequently, the degree of virulence has implications for the design of vaccines used to control streptococcosis. Initially, the vaccine, which was made in Indonesia to prevent streptococcosis, consisted of only one antigen, namely, *S. agalactiae* N14G from the island of Java. The next vaccine consisted of two antigens, namely, a combination of *S. agalactiae* and *Aeromonas hydrophila*, in which *A. hydrophila* was thought to often exacerbate Streptococcus. The next vaccine consists of three types of antigens with the aim providing of better protection against streptococcosis [76, 77].

Therefore, the level of virulence has an impact on the design of vaccines used to control streptococcosis. The virulent genes and their number are used as a basis for the selection of the bacteria that make up the vaccine. Vaccine designs have changed according to *S. agala*ctiae virulence in the field, from monovalent and bivalent to trivalent. In Indonesia, non-hemolytic *S. agalactiae* (biotype 2) N14G from West Java with 6/10 total virulence genes and β-hemolytic bacteria (biotype 1) NP105O and SG01-16 from Papua and Jambi with 7/10 virulent genes are potential candidates for the polyvalent vaccine design. The polyvalent vaccine composition was 30%:35%:35% in one preparation [78].

## Proposed Strategies

The future challenge in sustainable freshwater fish farming, both intensive and super-intensive, is to minimize the development of diseases and to reduce the use of antibiotics that jeopardize the health of humans, animals, and the environment through alternatives, such as vaccines, probiotics, prebiotics, synbiotics, natural ingredients (herbs), and immunostimulants.

In the future, especially for Indonesian aquaculture, polyvalent vaccine innovations using local isolates must continue to be developed to improve the coverage of pathogen diversity and cross-reactivity response between pathogens.

The development of polyvalent vaccines is more appropriate and designed based on the type and number of virulent genes from potential pathogens identified through biomolecular analysis and then combined among selected pathogen isolates from different regions.

There is a need for research that examines the use of microbial thermosensing by identifying the molecules that regulate it. Based on certain sequences and motifs, these molecules are used for the development of new vaccines or drugs.

## Conclusions and Prospects

The presence of microbial virulence, which leads to higher infectivity, is an adaptation of micro-organisms to environmental conditions that are different from normal. This condition is mediated by changes at the cell level to maintain normal microbial physiology and metabolism so that they can survive. Changes at the cellular level can be observed in terms of gene expression and the presence or absence of gene interference. It is necessary to consider manipulating pathogenic microbes or the environment so that the pathogen does not become more virulent and that the environment becomes stable.

Future research on the physiology of microbes evolving and adapting to extreme environments is required to develop strategies for controlling new emerging and re-emerging diseases that are harmful to humans, animals, and the environment.

## Authors’ Contributions

AML: Conceptualization, writing-original draft, writing-review and editing, visualization; AS, BRH, and SS: Validation, writing-review and editing; DC, MM, DS, DSy, and PES: Writing-review and editing. All authors have read, reviewed, and approved the final manuscript.
